# Supplementary figures and images for: A Cross-Sectional Study of the Associations between Chronotype, Social Jetlag and Subjective Sleep Quality in Healthy Adults
Source: Clocks Sleep. 2019 Dec 18;2(1):1–6. doi: 10.3390/clockssleep2010001 (PMC7445814; doi:10.3390/clockssleep2010001)

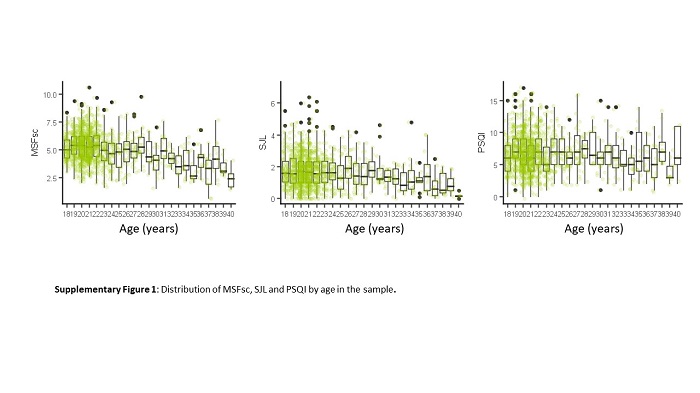

Supplement: Supplementary file 1 [file clockssleep-02-00001-s001.jpg]
